# Ozone in Epidemics

**Published:** 1855-09

**Authors:** Victor Meunier


Ozone in Epidemics.—[Translated from Revue de Thérapeutique Medico-Chirurgicale of May, 1855, for the New Orleans Med. and Sur. Journal, by Mrs. M. E. V.]

M. Wolf, director of the Observatory at Berne, announces in a letter addressed to the Academy of Sciences that, according to his personal observation, a rapid diminution of Ozone in the air is (if not always, at least usually) followed by a considerable augmentation of mortality.

What is Ozone? This is the question.

We answer with pleasure, for a new subject is in question, which is worthy attention under several aspects, and which recommends itself equally to the chemist, meteorologist, and physician. Henceforth the study of the variations of this new principle enters into daily meteorological observations under the same claim as that of temperature, atmospheric pressure, etc.

Ozone is nothing else than oxygen itself; this gas which enters for twenty-one centiémes (·21) in the composition of the air we breathe. But is oxygen so different from that to which chemists are accustomed to give that name, that they have a great deal of trouble to recognise it under its disguise.

For instance, oxygen is without scent, as every one knows; ozone on the contrary has a strong odor. It is by its scent, indeed, that its presence became known to observers; its name being taken from the Greek, reminds you of this property. Its odor oftentimes partakes of those of chlorine, of phosphorus, and of burning sulphur mixed with air. It manifests itself in the discharges attendant on the turning the plate of an electrical machine, or when there is a peal of thunder.

But the new and permanent qualities which oxygen acquires in becoming ozone are not confined to this; we know, for instance, that ordinary oxygen only combines slowly with mercury at the usual temperature; ozone, on the contrary, combines rapidly with this metal. In a word, the oxydizing qualities of Ozone are much more energetic than those of ordinary oxygen.

Van Marum was the first who found this remarkable substance.

It was in 1785; having at his disposal the great apparatus belonging to the Museum of Teyler, he excited sparks in a tube full of oxygen. A quarter of an hour afterwards, after 5,000 sparks, the oxygen had acquired a strong scent which he regarded as being very clearly that of electricity.

From 1785 to 1840, these remarkable experiments were completely lost sight of. In the course of the last year, M. de Schoenbein, Professor of Chemistry at Bale and inventor of the gun-cotton, decomposing water by means of an electrical battery, remarked that the production of hydrogen gas was accompanied by a very peculiar odor. He published a memoir on the subject. What was this new substance? Was it a simple element—was it an oxygenated product of azote or of hydrogen? The ingenious chemist left the question undecided, but he gave this odorous substance the name of *Ozone.*

In 1851, two *savants* of Geneva, MM. Marignac and De la Rive, concluded after a series of experiments that Ozone is nothing but oxygen in the peculiar state of chemical activity given to it by electricity. Berzelius and M. Faraday believed equally that there is here, an isomeric or allotropic state, that is to say, a simple modification of the oxygen. Finally in the same year, 1851, M. Schoenbein, who, on discussing this interesting question for the third time, agreed with MM. Marignac and La Rive.

Nevertheless, the majority of chemists admitted it hesitatingly. New experiments were necessary. Those made by MM. E. Frémy and Edmond Becquerel, in 1852, seemed to remove all doubts. They have shown, in confirmation of the experiments before mentioned, that electricity, acting on oxygen, developes new qualities in it; and these gentlemen have proposed changing the name of ozone into that of electrified oxygen (*Oygéne électrisé*).

Ozone then is nothing but a peculiar form of oxygen. Thus we are shown the changes that simple and compound substances experience in their most essential qualities from the force of exterior circumstances. The changes in oxygen that electricity causes in this case are comparable, in fact, to those produced by the solar rays on chlorine, whose affinities become much more energetic, and to those which heat developes in sulphur, phosphorus and carbon, whose colors, consistence, solubility and affinities they modify, and in many compound metallic oxides, for instance, experience under its influence isomeric transformations. Thus, this collection of mysterious facts is of powerful interest and fully studied, will doubtlessly modify the classic ideas already much shaken as to the pretended number of simple substances which are thus found numerous by combination.

Once admit that by the agency of electric sparks oxygen can enter into a state of chemical activity, then we must ask, if this change which we produce in our laboratories, is not produced spontaneously in the air; the more so as we cannot doubt that the atmosphere incessantly agitated by storms, experiences these chemical changes we speak of. This is a matter, however, of which one does not soon acquire a certainty.

Since 1850, M. Schoenbein has proved that Ozone decomposes the iodide of potassium, and he proved that a band of starched paper containing a small quantity of this salt, constitutes the most powerful, delicate reagent for discovering the presence of this new substance. A strip of paper prepared in this manner and exposed to the air, soon revealed, in changing from white, which was its original color before the experiment, to a blue of more or less intensity, the presence of ozone. That ozone then exists naturally in the air, is a point demonstrated; but does it always exist in the same proportions? The contrary is evident. Of what importance it is then, to study its variations? To do this it was necessary to bring back the observations to a common mode of comparison; nothing proved more easy.

Observers first arranged an ozonometric scale by dividing into a certain number of parts or degrees, the chromatic space comprised between the white which answers to the absence of ozone and the most intense blue that ozone can produce, at its maximum on ozonoscopic paper, putting on simple iodine. Ten was the number of visions adopted, and, this done, they possessed an *ozonoscope* by means of which they could measure the daily variations of the atmospheric ozone, as they could by means of the thermometer and barometer measure the daily changes of the temperature, and atmospheric pressure.

Several philosophers, knowing the importance of this new branch of meteorological observation. have pursued it. MM. Bœckel at Strasburg, Simonin, sen., at Nancy; Wolf, at Beane; Billiard, at Corbigny; Schapter and Besluber, in Germany; Gailliard, in America, etc., are among these observers.

So that here is a substance, whose existence was not suspected a few years ago, and one which acts on us and on all animated nature. How is it possible to doubt as to the intensity of its action? How can one doubt but that considerable variations in the oxydizing power of respirable gas have a powerful influence on respiration, and, in consequence, on all the vital functions?

An American physician, M. E. S. Gaillard, finds a connection between ozone in atmospheric air and the appearance of intermittent fevers.

According to Dr. Bœckel, malaria always shows itself with the zero of the Ozonoscope, and the same thing takes place when paludal fevers prevail.

According to M. Schoenbein, a considerable quantity of ozone was observed in the air of Berlin during the epidemic *Grippe* and during a medical constitution of the air predisposing to affections of the chest; and the reverse took place under the prevalence of the gastric constitution.

According to the same observer, ozone was completely wanting in the atmosphere of the same city during a cholera epidemic.

According to Mr. Bœckel, the same thing occurred at Strasburg. The appearance of the cholera coincided with the absence of ozone, and the ozone reappeared as the cholera decreased.

M. Billiard regards the diminution of ozone as the first cause of this terrible malady.

Finally, without going so far, Mr. Wolf, in the letter which occasioned this article, confirms, as to the city of Berne in which he resides, the observations made at Strasburg by Dr. Bœckel.

It appears likely to us that ozone has not only great physiological parts to perform, but it is necessary to employ it in explaining several physical and chemical phenomena, such for instance, as the productions of nitric acid in the atmosphere, and the disengagement of the odor which so often accompanies thunder.

(*L'Ami des Sciences.*) Victor Meunier.

---

[Addendum, from the *Gaz. Hebdon. de Med.* of May 11, 1855.]

Ozone and Cholera.—Having procured the daily list of deaths from cholera at Aarau, in Switzerland, from the 15th of August to the 14th, of October, 1854, M. Wolf, director of the observatory at Berne, has classed the days in which no deaths occured—those in which there were one or two, and finally those in which there were three or more: "I have found," says he, "that the corresponding mean of the actions of ozone at Berne is—

| | | |
|---|---|---|
| For the days of the first class | - - - | 6. 48; |
| " second class | - - - | 5. 48; |
| " third class | - - - | 4. 58; |

"I conclude from this," says M. Wolf, "that the cholera, to say the least, is very much favored by the diminution of ozone.—*L'Ami des Sciences.*

